# Supplementary material for: Evidence supported by Mendelian randomization: impact on inflammatory factors in knee osteoarthritis
Source: Front Med (Lausanne). 2024 May 28;11:1382836. doi: 10.3389/fmed.2024.1382836 (PMC11165061; doi:10.3389/fmed.2024.1382836)
Supplement: Supplementary file 1 [file Data_Sheet_1.ZIP › data/GCST90274789_5e6/789.funnel_plot.pdf]

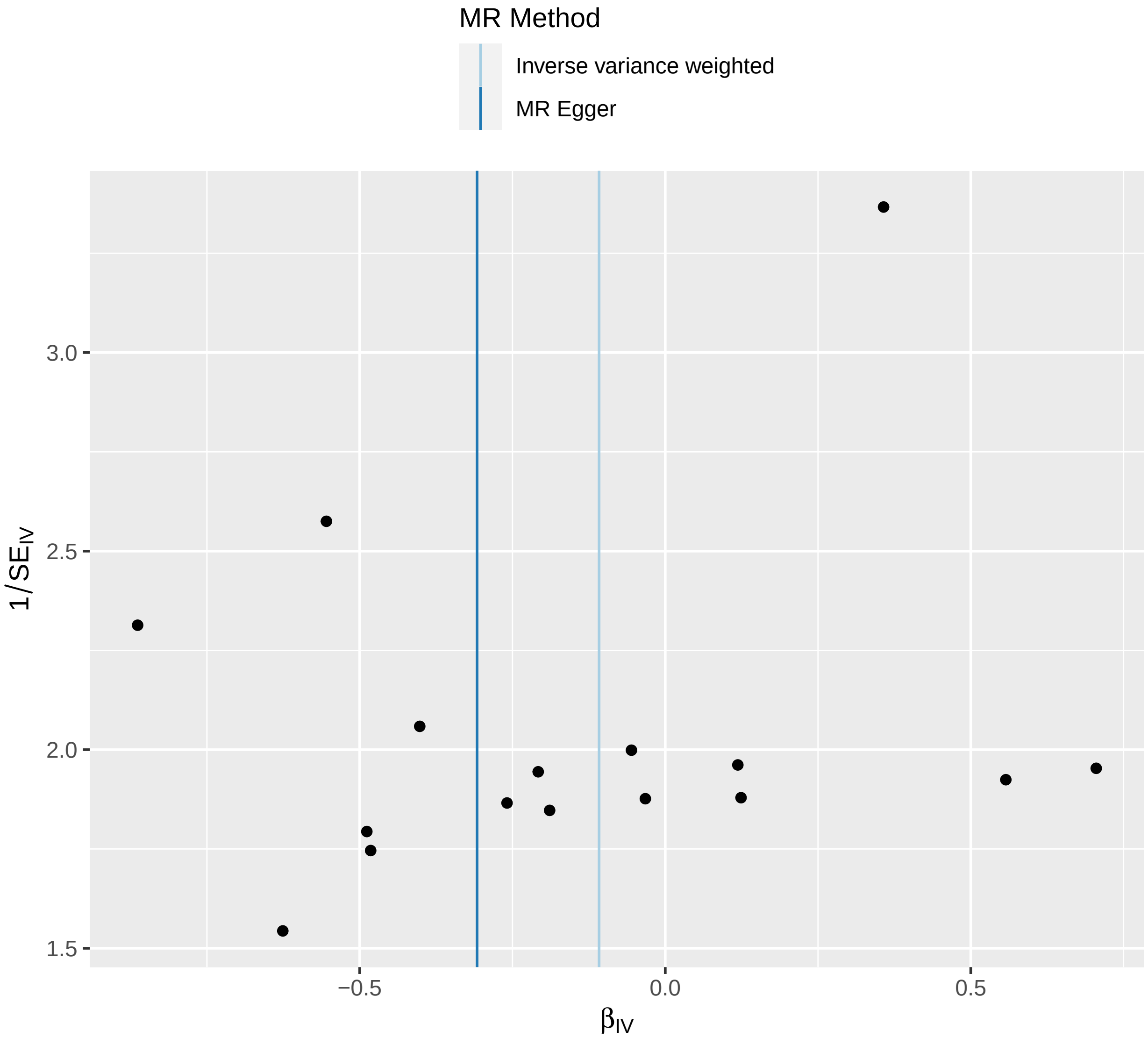
MR Method
Inverse variance weighted
MR Egger
3.0
2.5
2.0
1.5
1/SE_IV
−0.5
0.0
0.5
β_IV
